# Editorial: Pandemic preparedness in vaccine safety and regulation

**DOI:** 10.3389/fdsfr.2026.1824880

**Published:** 2026-04-14

**Authors:** Manal M. Younus, Florence van Hunsel

**Affiliations:** 1 Iraqi Pharmacovigilance Centre, Ministry of Health, Baghdad, Iraq; 2 Netherlands Pharmacovigilance Centre Lareb, sHertogenbosch, Netherlands; 3 Department of PharmacoTherapy, Epidemiology and Economics, Groningen Research Institute of Pharmacy (GRIP), University of Groningen, Groningen, Netherlands

**Keywords:** COVID-19 pandemic, ethics, pharmacovigilance, regulatory processes, SARS-COV-2 virus, signal detection, therapeutic development, trust

## 1 Introduction

Editorial on the *Research Topic Pandemic Preparedness in Vaccine Safety and Regulation*. The COVID-19 pandemic, caused by the SARS-CoV-2 virus, led to a global health crisis (Pollard et al., 2020). Vaccines for the prevention of COVID-19 were developed at a rapid speed and deployed on an unprecedented scale (Burgos et al., 2021). Monitoring the safety of these vaccines in real-world circumstances after marketing authorization was therefore of great importance. Even though the World Health Organization (WHO) declared the end of COVID-19 as a public health emergency on 5 May 2023 (Sarker et al., 2023), preparedness for a future pandemic remains essential. Sharing experiences gained from safety monitoring efforts during the COVID-19 pandemic provides valuable insights into challenges, successes, and potential solutions that can strengthen preparedness for any emerging health crisis.

In this editorial, we highlight the lessons learned from the ten studies submitted to this Research Topic. The main points of these articles are grouped into five thematic areas.

## 2 Pharmacovigilance infrastructure and signal detection

The Dutch national pharmacovigilance center Lareb processed more than 233,000 individual case safety reports (ICSRs) related to COVID-19 vaccines, during the pandemic, largely from vaccinated people themselves. The volume of the reports was around eight times its normal annual intake, while simultaneously running a cohort event monitoring study with over 27,000 participants. Three systemic challenges became apparent. The sheer scale of incoming reports overwhelmed manual processing capacity, and treatment-related reports were paradoxically sparce: only 265 ICSRs were received for COVID-19 therapies, likely because healthcare workers were already stretched beyond capacity. Data infrastructure created a second bottleneck as timely linkage between the national vaccination registry with the spontaneous reporting system and health records was lacking in early 2021 when the vaccination campaigns started in the Netherlands. Third, public communication demands exploded, with website visits rising 522% in 2021 alone. The authors call for pre-established data linkage agreements, investment in artificial intelligence for automation in ICSR processing, including triage of ICSRs, and communication capacity built to scale before the next emergency (van Hunsel and Kant, 2025).

Two complementary papers produced through the “Beyond COVID-19 Monitoring Excellence” (BeCOME) initiative examine the specific challenge of observed-to-expected (O/E) analyses. These analyses compare post-vaccination adverse events of special interest (AESI) rates against expected baseline rates and were mandated by the European Medicines Agency (EMA) for primarily signal detection during the pandemic, an unusual departure from their traditional role in signal refinement. Despite enormous resources investment, with some manufacturers conducting over 600 O/E calculations per reporting cycle by late 2023, no *de novo* safety signals were detected through this method. O/E did prove valuable in its traditional role, stratified analyses helped characterize the elevated myocarditis risk in young males after mRNA vaccination, but its use as a primary detector was undermined by unreliable numerators (inflated by unadjusted cases and stimulated media reporting) and unreliable denominators (background incidence rates that were inconsistent, late or simply unavailable for novel conditions like TTS). The authors of both papers call for O/E to be returned to its established role as a refinement tool with multi-stakeholders agreement on harmonized methodology secured in advance of future emergencies (Serradell et al.; Gandhi-Banga et al.).

The BeCOME initiative also addresses the background incidence rate (BIR) problem directly. Five major BIR generating initiatives operated during the pandemic, in Europe, the United States, and across multinational collaborations, yet there were issues with usability of data when vaccines first rolled out in December 2020. BIRs for the same condition varied by up to six-folds across databases, case definitions were frequently unvalidated, and data for subpopulations including pregnant women, minority ethnic groups, and paediatric age strata were almost entirely absent. For low and middle income countries (LMICs), where many outbreaks originate, BIRs were essentially not existent. The paper calls for sustainable, regularly updated BIR generation mechanisms with harmonized case definitions and proactive subpopulation data development to be established well before the next pandemic (Gandhi-Banga et al.).

## 3 Medical coding: MedDRA’s pandemic response

The international medical terminology standard MedDRA, maintained by the Maintenance and Support Services Organization (MSSO) under International Council for Harmonization of Technical Requirements for Pharmaceuticals for human Use (ICH) oversight, plays a foundational role in pharmacovigilance by enabling consistent adverse event coding across databases, regulatory systems, and national boundaries. When COVID-19 emerged, MedDRA’s standard 6-month release cycle was incompatible with the emergency of the situation. In April 2020, the MSSO issued an unprecedented out of cycle release including 68 new COVID-19 terms, and subsequent releases built iteratively on this, adding terminology for long COVID, vaccine efficacy and failure, variant-specific outcomes, and immune response monitoring through to version 26.0 in March 2023. Collaboration with the WHO and the Uppsala Monitoring Center (UMC) enabled standardized adverse events following immunization (AEFI) reporting through the VigiFlow platform, and existing Standardized MedDRA Queries were repurposed to detect signals including TTS. The pandemic both demonstrated MedDRA’s adaptability and revealed that cross-regional coordination across regulatory systems with different timelines and readiness levels remained a meaningful constraint on rapid adoption of updated terminology (O'Hare et al.).

## 4 Regulatory processes and therapeutic development

Katayama and Shikano systematically analyzed the fourteen COVID-19 therapeutics that received US Emergency Use Authorization (EUA), examining how development timelines ranging from 92 to 1,154 days were achieved and what lessons can be generalized. The single most powerful accelerant was the availability of previous clinical data: products such as remdesivir, baricitinib, and tocilizumab that had existing evidence from other indications required a medium of 1.5 new studies to support authorization, compared with 6.0 for genuinely novel agents. Only remdesivir met the target of the 100 Days Mission, and only by drawing on data already accumulated for other viral infections. Additional accelerating strategies included parallel and non-sequential phase development, adaptive protocol design, dose determination from non-clinical data, and interim analysis submissions. The authors argue that the most durable lesson is that the framework for applying regulatory flexibilities, cataloguing repurposable candidates, pre-identifying global trial site networks, and clarifying regulatory conditions in advance, must be assembled before, not during, an emergency (Katayama and Shikano).

A related BeCOME policy review examines post-approval vaccine safety studies in pregnant populations and finds that of 46 completed studies, only two led to a product label update, while primary data Research Topic studies averaged 7.5 years to completion. Five existing guidance documents from the FDA and EMA were identified, but none contains vaccine-specific recommendations; guidance is oriented toward pharmaceutical drugs and leaves manufacturers without clear expectations when designing pregnancy research for vaccine products. Eight specific gaps are identified, covering study design selection, comparator group specification, vaccine exposure characterization, harmonized prioritized outcomes list, sample size targets tied to pre-defined minimal detectable risks, study duration and stopping rules, multi-sponsor registry frameworks, and guidance on rapid cycle analyses. Harmonising and expanding regulatory guidance specifically for vaccine pregnancy research would accelerate evidence generation and better protect a population systematically underrepresented in pre-approval trials (MacDonald et al.).

## 5 Ethics, trust and vaccine acceptance

The inter-company Ethics Committee of Turin reviewed 1,667 protocols between 2020 and 2022, with COVID-19 related submissions concentrated heavily in the early months of the pandemic. Three statistically significant differences distinguished COVID-19 from non-COVID research: COVID-19 studies were overwhelmingly observational and not-for-profit, and drew disproportionately from frontline specialties including critical care, infectious diseases, and geriatric medicine. The authors identify the core tension that ethics committee navigated, the dual obligation to expedite approvals for urgent research while maintaining the rigor that protects scientific validity and participant rights, and conclude that ethics committees must be understood as enabling structures rather than administrative obstacles. They call for international standardization of expected review procedures so that ethical standards remain non-negotiable regardless of external pressure (Tattoli et al.).

A Romanian study developed and validated the RO-CVH scale, a 15-item instrument measuring COVID-19 vaccine hesitancy among industrial workers, identifying three distinct hesitancy dimensions: confidence in vaccine information, beliefs about safety and efficacy, and the perception of vaccination as an instrument of population control. That 73.4% of the validation sample believed COVID-19 was artificially created, illustrates the depth of the misinformation challenge this instrument is designed to address (Turcu-S et al.). A cross-sectional survey conducted in Central Texas in the first week of vaccine availability for the general public adds complementary evidence among 654 (Affordable Care Act) ACA-insured respondents, the strongest independent predictor of intending to vaccinate was believing the vaccine would be personally protective, conferring 13-folds higher odds, followed closely by believing it was safe. Transparency of the authorization process was also independently significant, while concerns about the speed of authorization was not, suggesting that institutional trust matters more than timeline. Both studies converge on the message that targeted communication addressing perceived safety, effectiveness, and process integrity is as essential a component of pandemic preparedness as the vaccines themselves (Lopez Bray et al.).

## 6 The 100 days mission and systemic readiness

A global expert survey on the feasibility of Coalition for Epidemic Preparedness Innovations (CEPI)’s 100 Days Mission, the ambition to develop, authorize, and deploy effective vaccines within 100 days of identifying a pandemic pathogen, found that high income countries (HIC) experts were predominantly skeptical of success (57.9% anticipated failure) while low and LMICs experts were modestly more optimistic. All 85 respondents agreed that political will, sustainable financing, and stakeholders collaboration are the most critical prerequisites, with two-thirds identifying funding instability as the single greatest obstacle. Surveillance systems, vaccine prototype, vaccine candidate libraries, and clinical trial networks were identified as feasible near-term priorities, while global biomanufacturing capacity and early biological markers were considered far harder to deliver quickly. The survey reinforces the cross-cutting message that runs through all ten papers, the most important investments in pandemic preparedness are those that build and sustain infrastructure continuously between emergencies; warm, functional, and ready to scale, rather than those assembled only in response to a crisis already underway (Pencelli et al.).

## 7 Conclusion

The collective evidence assembled in this Research Topic points to a clear transition: pandemic preparedness must be approached as system design, not as a reactive, episodic response. COVID-19 demonstrated that historical models of vaccine safety and regulation that are dependent on delayed data linkage, fragmented methodologies, and *ad hoc* scale up, are no longer tenable at the speed and scale of modern vaccine deployment. In their place, a paradigm of continuous, anticipatory readiness is emerging, anchored in real-time data Research Topic and linkage, resilient pharmacovigilance capacity, rapid and methodologically disciplined signal detection, and regulatory action that is adaptive, coordinated, and transparent. The next crisis will not wait for our processes to catch up; preparedness must therefore be built as a standing capability, kept warm between emergencies, ready to accelerate without compromising scientific rigor, ethics, or trust.
